# Bis[1-phenyl-3-(1*H*-1,2,4-triazol-1-yl-κ*N* ^4^)propan-1-one]bis­(thio­cyanato-κ*N*)copper(II)

**DOI:** 10.1107/S160053681203108X

**Published:** 2012-07-25

**Authors:** Hua Cai, Ying Guo, Jian-Gang Li

**Affiliations:** a College of Science, Civil Aviation University of China, Tianjin 300300, People’s Republic of China

## Abstract

The title compound, [Cu(NCS)_2_(C_11_H_11_N_3_O)_2_], contains two independent Cu^II^ atoms. Each Cu^II^ atom, lying on an inversion center, is coordinated by two N atoms from two NCS^−^ anions and two N atoms from two monodentate 1-phenyl-3-(1*H*-1,2,4-triazol-1-yl)propan-1-one ligands in a distorted square-planar geometry. Two S atoms from adjacent mol­ecules occupy the axial positions with long Cu⋯S distances [3.0495 (10) and 3.1045 (9) Å] and complete the overall distorted octahedral coordination sphere. Weak inter­molecular C—H⋯O hydrogen bonds are present.

## Related literature

For related structures, see: Guo & Cai (2007 ▶); Yue *et al.* (2008 ▶).
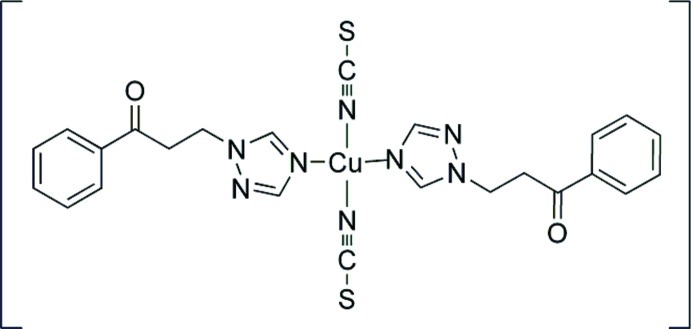


## Experimental

### 

#### Crystal data

[Cu(NCS)_2_(C_11_H_11_N_3_O)_2_]
*M* *_r_* = 582.16
Triclinic, 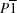

*a* = 9.8643 (8) Å
*b* = 10.1267 (9) Å
*c* = 14.3538 (12) Å
α = 91.149 (1)°
β = 101.270 (1)°
γ = 110.857 (1)°
*V* = 1307.75 (19) Å^3^
*Z* = 2
Mo *K*α radiation
μ = 1.03 mm^−1^
*T* = 293 K
0.28 × 0.24 × 0.16 mm

#### Data collection

Bruker APEXII CCD diffractometer
Absorption correction: multi-scan (*SADABS*; Sheldrick, 1996 ▶) *T*_min_ = 0.749, *T*_max_ = 0.848
7171 measured reflections
4559 independent reflections
3860 reflections with *I* > 2σ(*I*)
*R*_int_ = 0.013

#### Refinement

*R*[*F* ^2^ > 2σ(*F* ^2^)] = 0.031
*wR*(*F* ^2^) = 0.091
*S* = 1.08
4559 reflections
337 parameters
H-atom parameters constrained
Δρ_max_ = 0.52 e Å^−3^
Δρ_min_ = −0.41 e Å^−3^

### 

Data collection: *APEX2* (Bruker, 2007 ▶); cell refinement: *SAINT* (Bruker, 2007 ▶); data reduction: *SAINT*; program(s) used to solve structure: *SHELXS97* (Sheldrick, 2008 ▶); program(s) used to refine structure: *SHELXL97* (Sheldrick, 2008 ▶); molecular graphics: *XP* in *SHELXTL* (Sheldrick, 2008 ▶); software used to prepare material for publication: *SHELXTL*.

## Supplementary Material

Crystal structure: contains datablock(s) global, I. DOI: 10.1107/S160053681203108X/hy2568sup1.cif

Structure factors: contains datablock(s) I. DOI: 10.1107/S160053681203108X/hy2568Isup2.hkl

Additional supplementary materials:  crystallographic information; 3D view; checkCIF report

## Figures and Tables

**Table 1:** Hydrogen-bond geometry (Å, °)

*D*—H⋯*A*	*D*—H	H⋯*A*	*D*⋯*A*	*D*—H⋯*A*
C13—H13⋯O1	0.93	2.58	3.330 (3)	138

**Fig. 1.**
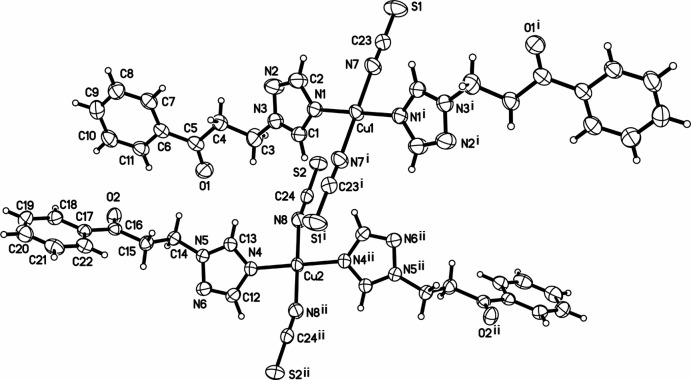
The molecular structure of the title compound, showing the 50% probability ellipsoids. [Symmetry codes: (i) -x, 1-y, -z; (ii) 1-x, -y, -z.]

## supplementary crystallographic information

### Comment

Pseudohalide anions N3^-^, NCS^-^ and NCO^-^ are known as extremely versatile ligands in coordination chemistry because of their multiple bridging modes (Yue *et al.*, 2008). Recently, we have initiated a research program of synthesizing supermolecules based on pseudohalide and flexible ligands that consist of a propanone unit substituted with an imidazole and a phenyl group (Guo & Cai, 2007). To further explore this series, we synthesized the title compound, a new Cu^II^ complex based on the mixed ligands, thiocyanato and 3-(1*H*-1,2,4-triazol-1-yl)-1-phenylpropan-1-one (*L*) which consists of a propanone unit substituted with a triazole and a phenyl group. In the mononuclear title complex (Fig. 1), each Cu^II^ atom is four-coordinated by two monodentate *L* ligands and two NCS^-^ anions, forming a square-planar geometry. Weak intermolecular C—H···O hydrogen bonds are present (Table 1).

### Experimental

NH_4_SCN (15.2 mg, 0.2 mmol) was added into an acetonitrile solution of *L* (25.6 mg, 0.1 mmol) with stirring. The acetonitrile solution was added into a solution of CuCl_2_.2H_2_O (17.0 mg, 0.1 mmol) in acetonitrile/H_2_O (10 ml, v/v 1:1) with vigorous stirring for *ca* 30 min. The reaction solution was filtered and left to stand at room temperature. Blue block crystals of the title compound suitable for X-ray analysis were obtained in 65% yield by slow evaporation of the solvent over a period of 1 week. Analysis, calculated for Cu_2_C_48_H_44_N_16_O_4_S_4_: C 49.52, H 3.81, N 19.25%; found: C 49.45, H 3.89, N 19.36%.

### Refinement

Although all H atoms were visible in difference maps, they were finally placed in geometrically calculated positions, with C—H = 0.93 (aromatic) and 0.97 (CH_2_) Å, and refined as riding atoms, with *U*_iso_(H) = 1.2*U*_eq_(C).

### Crystal data

**Table:**

[Cu(NCS)_2_(C_11_H_11_N_3_O)_2_]	*Z* = 2
*M**_r_* = 582.16	*F*(000) = 598
Triclinic, *P*1	*D*_x_ = 1.479 Mg m^−^^3^
Hall symbol: -P 1	Mo *K*α radiation, λ = 0.71073 Å
*a* = 9.8643 (8) Å	Cell parameters from 3208 reflections
*b* = 10.1267 (9) Å	θ = 2.5–27.7°
*c* = 14.3538 (12) Å	µ = 1.03 mm^−^^1^
α = 91.149 (1)°	*T* = 293 K
β = 101.270 (1)°	Block, blue
γ = 110.857 (1)°	0.28 × 0.24 × 0.16 mm
*V* = 1307.75 (19) Å^3^	

### Data collection

**Table:**

Bruker APEXII CCD diffractometer	4559 independent reflections
Radiation source: fine-focus sealed tube	3860 reflections with *I* > 2σ(*I*)
Graphite monochromator	*R*_int_ = 0.013
φ and ω scans	θ_max_ = 25.0°, θ_min_ = 2.2°
Absorption correction: multi-scan (*SADABS*; Sheldrick, 1996)	*h* = −10→11
*T*_min_ = 0.749, *T*_max_ = 0.848	*k* = −10→12
7171 measured reflections	*l* = −15→17

### Refinement

**Table:**

Refinement on *F*^2^	Primary atom site location: structure-invariant direct methods
Least-squares matrix: full	Secondary atom site location: difference Fourier map
*R*[*F*^2^ > 2σ(*F*^2^)] = 0.031	Hydrogen site location: inferred from neighbouring sites
*wR*(*F*^2^) = 0.091	H-atom parameters constrained
*S* = 1.08	*w* = 1/[σ^2^(*F*_o_^2^) + (0.0456*P*)^2^ + 0.6431*P*] where *P* = (*F*_o_^2^ + 2*F*_c_^2^)/3
4559 reflections	(Δ/σ)_max_ < 0.001
337 parameters	Δρ_max_ = 0.52 e Å^−^^3^
0 restraints	Δρ_min_ = −0.41 e Å^−^^3^

### Special details

**Table:**

Geometry. All e.s.d.'s (except the e.s.d. in the dihedral angle between two l.s. planes) are estimated using the full covariance matrix. The cell e.s.d.'s are taken into account individually in the estimation of e.s.d.'s in distances, angles and torsion angles; correlations between e.s.d.'s in cell parameters are only used when they are defined by crystal symmetry. An approximate (isotropic) treatment of cell e.s.d.'s is used for estimating e.s.d.'s involving l.s. planes.
Refinement. Refinement of *F*^2^ against ALL reflections. The weighted *R*-factor *wR* and goodness of fit *S* are based on *F*^2^, conventional *R*-factors *R* are based on *F*, with *F* set to zero for negative *F*^2^. The threshold expression of *F*^2^ > σ(*F*^2^) is used only for calculating *R*-factors(gt) *etc*. and is not relevant to the choice of reflections for refinement. *R*-factors based on *F*^2^ are statistically about twice as large as those based on *F*, and *R*- factors based on ALL data will be even larger.

### Fractional atomic coordinates and isotropic or equivalent isotropic displacement parameters (Å^2^)

**Table:**

	*x*	*y*	*z*	*U*_iso_*/*U*_eq_	
Cu1	0.0000	0.5000	0.0000	0.03694 (13)	
Cu2	0.5000	0.0000	0.0000	0.04017 (13)	
S1	−0.48000 (8)	0.29758 (9)	0.06142 (7)	0.0654 (2)	
S2	0.06035 (8)	−0.21058 (9)	0.10990 (6)	0.0600 (2)	
O1	0.4874 (2)	0.1466 (2)	0.35980 (13)	0.0532 (5)	
O2	0.87681 (19)	0.03667 (17)	0.56084 (12)	0.0431 (4)	
N1	0.0706 (2)	0.4157 (2)	0.11707 (14)	0.0378 (5)	
N2	0.0832 (3)	0.3365 (3)	0.26177 (17)	0.0553 (6)	
N3	0.1774 (2)	0.3051 (2)	0.21618 (14)	0.0397 (5)	
N4	0.6443 (2)	0.0025 (2)	0.11758 (14)	0.0389 (5)	
N5	0.7402 (2)	−0.0004 (2)	0.26570 (13)	0.0349 (4)	
N6	0.8416 (2)	−0.0119 (3)	0.21731 (15)	0.0511 (6)	
N7	−0.1992 (2)	0.4382 (2)	0.02640 (15)	0.0429 (5)	
N8	0.3355 (2)	−0.0705 (2)	0.06659 (15)	0.0470 (5)	
C1	0.1693 (3)	0.3532 (3)	0.13169 (17)	0.0419 (6)	
H1	0.2249	0.3445	0.0882	0.050*	
C2	0.0219 (3)	0.4019 (3)	0.19914 (19)	0.0530 (7)	
H2	−0.0498	0.4363	0.2103	0.064*	
C3	0.2643 (3)	0.2222 (3)	0.25970 (19)	0.0491 (6)	
H3A	0.3470	0.2360	0.2291	0.059*	
H3B	0.2023	0.1221	0.2499	0.059*	
C4	0.3223 (3)	0.2669 (3)	0.36542 (17)	0.0398 (6)	
H4A	0.3629	0.3698	0.3756	0.048*	
H4B	0.2403	0.2330	0.3976	0.048*	
C5	0.4406 (3)	0.2112 (2)	0.40930 (18)	0.0366 (5)	
C6	0.5010 (2)	0.2403 (2)	0.51463 (17)	0.0346 (5)	
C7	0.4606 (3)	0.3234 (3)	0.57345 (19)	0.0425 (6)	
H7	0.3920	0.3636	0.5475	0.051*	
C8	0.5217 (3)	0.3471 (3)	0.6704 (2)	0.0482 (6)	
H8	0.4929	0.4020	0.7094	0.058*	
C9	0.6246 (3)	0.2897 (3)	0.7096 (2)	0.0499 (7)	
H9	0.6665	0.3070	0.7747	0.060*	
C10	0.6655 (3)	0.2065 (3)	0.6520 (2)	0.0495 (7)	
H10	0.7351	0.1677	0.6782	0.059*	
C11	0.6040 (3)	0.1811 (3)	0.55633 (18)	0.0421 (6)	
H11	0.6310	0.1234	0.5183	0.050*	
C12	0.7786 (3)	−0.0106 (3)	0.12931 (18)	0.0486 (7)	
H12	0.8219	−0.0181	0.0784	0.058*	
C13	0.6252 (3)	0.0090 (3)	0.20563 (16)	0.0372 (5)	
H13	0.5429	0.0188	0.2228	0.045*	
C14	0.7671 (3)	0.0026 (3)	0.36889 (16)	0.0360 (5)	
H14A	0.7983	−0.0751	0.3887	0.043*	
H14B	0.6758	−0.0100	0.3897	0.043*	
C15	0.8855 (3)	0.1414 (2)	0.41492 (16)	0.0364 (5)	
H15A	0.9720	0.1595	0.3874	0.044*	
H15B	0.8488	0.2175	0.4011	0.044*	
C16	0.9308 (2)	0.1423 (2)	0.52113 (16)	0.0314 (5)	
C17	1.0427 (2)	0.2750 (2)	0.57691 (16)	0.0316 (5)	
C18	1.0867 (3)	0.2771 (3)	0.67518 (17)	0.0398 (6)	
H18	1.0451	0.1966	0.7053	0.048*	
C19	1.1913 (3)	0.3976 (3)	0.72822 (19)	0.0496 (7)	
H19	1.2211	0.3979	0.7938	0.060*	
C20	1.2523 (3)	0.5184 (3)	0.6840 (2)	0.0528 (7)	
H20	1.3223	0.6000	0.7201	0.063*	
C21	1.2093 (3)	0.5178 (3)	0.5862 (2)	0.0490 (7)	
H21	1.2502	0.5992	0.5568	0.059*	
C22	1.1058 (3)	0.3970 (3)	0.53216 (19)	0.0399 (6)	
H22	1.0781	0.3964	0.4664	0.048*	
C23	−0.3161 (3)	0.3805 (3)	0.03986 (17)	0.0383 (5)	
C24	0.2211 (3)	−0.1283 (3)	0.08425 (16)	0.0389 (6)	

### Atomic displacement parameters (Å^2^)

**Table:**

	*U*^11^	*U*^22^	*U*^33^	*U*^12^	*U*^13^	*U*^23^
Cu1	0.0272 (2)	0.0509 (3)	0.0339 (2)	0.01463 (18)	0.00743 (16)	0.01850 (18)
Cu2	0.0257 (2)	0.0606 (3)	0.0262 (2)	0.00673 (19)	0.00434 (16)	0.01022 (18)
S1	0.0396 (4)	0.0621 (5)	0.0905 (6)	0.0055 (3)	0.0312 (4)	−0.0025 (4)
S2	0.0370 (4)	0.0830 (5)	0.0544 (4)	0.0093 (4)	0.0220 (3)	0.0063 (4)
O1	0.0587 (12)	0.0689 (13)	0.0468 (11)	0.0412 (10)	0.0110 (9)	0.0079 (9)
O2	0.0478 (10)	0.0405 (9)	0.0342 (9)	0.0092 (8)	0.0059 (8)	0.0096 (7)
N1	0.0362 (11)	0.0484 (12)	0.0335 (11)	0.0195 (9)	0.0093 (9)	0.0135 (9)
N2	0.0566 (15)	0.0827 (17)	0.0481 (13)	0.0430 (13)	0.0246 (11)	0.0305 (12)
N3	0.0417 (12)	0.0471 (12)	0.0368 (11)	0.0239 (10)	0.0080 (9)	0.0110 (9)
N4	0.0322 (10)	0.0539 (13)	0.0287 (10)	0.0148 (9)	0.0040 (8)	0.0041 (9)
N5	0.0339 (10)	0.0441 (11)	0.0282 (10)	0.0166 (9)	0.0058 (8)	0.0038 (8)
N6	0.0455 (13)	0.0842 (17)	0.0347 (12)	0.0363 (12)	0.0103 (10)	0.0066 (11)
N7	0.0316 (11)	0.0507 (12)	0.0449 (12)	0.0125 (10)	0.0092 (9)	0.0139 (10)
N8	0.0356 (12)	0.0636 (14)	0.0350 (11)	0.0089 (10)	0.0086 (9)	0.0146 (10)
C1	0.0421 (14)	0.0592 (16)	0.0326 (13)	0.0273 (13)	0.0097 (11)	0.0109 (11)
C2	0.0566 (17)	0.083 (2)	0.0437 (15)	0.0475 (16)	0.0212 (13)	0.0292 (14)
C3	0.0595 (17)	0.0524 (16)	0.0441 (15)	0.0351 (14)	0.0031 (13)	0.0099 (12)
C4	0.0398 (13)	0.0432 (14)	0.0411 (14)	0.0208 (11)	0.0077 (11)	0.0132 (11)
C5	0.0314 (12)	0.0349 (12)	0.0463 (14)	0.0131 (10)	0.0116 (11)	0.0139 (10)
C6	0.0292 (12)	0.0348 (12)	0.0418 (13)	0.0126 (10)	0.0098 (10)	0.0140 (10)
C7	0.0386 (13)	0.0413 (14)	0.0508 (15)	0.0190 (11)	0.0076 (11)	0.0102 (11)
C8	0.0498 (16)	0.0441 (15)	0.0499 (16)	0.0176 (12)	0.0085 (13)	0.0001 (12)
C9	0.0457 (15)	0.0522 (16)	0.0431 (15)	0.0118 (13)	0.0013 (12)	0.0059 (12)
C10	0.0400 (14)	0.0588 (17)	0.0522 (16)	0.0237 (13)	0.0039 (12)	0.0136 (13)
C11	0.0383 (14)	0.0491 (15)	0.0455 (15)	0.0222 (12)	0.0116 (11)	0.0126 (11)
C12	0.0466 (15)	0.0775 (19)	0.0302 (13)	0.0323 (14)	0.0097 (11)	0.0047 (12)
C13	0.0321 (12)	0.0490 (14)	0.0308 (12)	0.0154 (11)	0.0059 (10)	0.0064 (10)
C14	0.0366 (13)	0.0448 (13)	0.0275 (12)	0.0157 (11)	0.0070 (10)	0.0069 (10)
C15	0.0399 (13)	0.0397 (13)	0.0296 (12)	0.0143 (11)	0.0077 (10)	0.0082 (10)
C16	0.0306 (12)	0.0372 (12)	0.0319 (12)	0.0178 (10)	0.0087 (9)	0.0078 (10)
C17	0.0303 (12)	0.0354 (12)	0.0339 (12)	0.0170 (10)	0.0087 (9)	0.0036 (9)
C18	0.0411 (14)	0.0434 (14)	0.0386 (14)	0.0188 (11)	0.0109 (11)	0.0018 (11)
C19	0.0485 (16)	0.0588 (17)	0.0396 (14)	0.0210 (13)	0.0045 (12)	−0.0120 (12)
C20	0.0409 (15)	0.0437 (15)	0.068 (2)	0.0114 (12)	0.0084 (13)	−0.0138 (13)
C21	0.0400 (14)	0.0375 (14)	0.071 (2)	0.0130 (12)	0.0187 (13)	0.0059 (13)
C22	0.0359 (13)	0.0403 (13)	0.0466 (14)	0.0163 (11)	0.0111 (11)	0.0085 (11)
C23	0.0367 (14)	0.0402 (13)	0.0372 (13)	0.0137 (11)	0.0066 (10)	0.0031 (10)
C24	0.0365 (14)	0.0503 (14)	0.0264 (12)	0.0132 (12)	0.0040 (10)	0.0059 (10)

### Geometric parameters (Å, º)

**Table:**

Cu1—N7^i^	1.955 (2)	C4—H4B	0.9700
Cu1—N7	1.955 (2)	C5—C6	1.494 (3)
Cu1—N1^i^	2.0150 (18)	C6—C7	1.387 (3)
Cu1—N1	2.0150 (18)	C6—C11	1.400 (3)
Cu2—N8^ii^	1.966 (2)	C7—C8	1.383 (4)
Cu2—N8	1.966 (2)	C7—H7	0.9300
Cu2—N4	1.9771 (19)	C8—C9	1.377 (4)
Cu2—N4^ii^	1.9771 (19)	C8—H8	0.9300
S1—C23	1.629 (3)	C9—C10	1.379 (4)
S2—C24	1.626 (3)	C9—H9	0.9300
O1—C5	1.213 (3)	C10—C11	1.367 (4)
O2—C16	1.221 (3)	C10—H10	0.9300
N1—C1	1.325 (3)	C11—H11	0.9300
N1—C2	1.348 (3)	C12—H12	0.9300
N2—C2	1.307 (3)	C13—H13	0.9300
N2—N3	1.355 (3)	C14—C15	1.506 (3)
N3—C1	1.315 (3)	C14—H14A	0.9700
N3—C3	1.466 (3)	C14—H14B	0.9700
N4—C13	1.318 (3)	C15—C16	1.501 (3)
N4—C12	1.356 (3)	C15—H15A	0.9700
N5—C13	1.318 (3)	C15—H15B	0.9700
N5—N6	1.359 (3)	C16—C17	1.490 (3)
N5—C14	1.451 (3)	C17—C18	1.390 (3)
N6—C12	1.297 (3)	C17—C22	1.402 (3)
N7—C23	1.149 (3)	C18—C19	1.375 (4)
N8—C24	1.152 (3)	C18—H18	0.9300
C1—H1	0.9300	C19—C20	1.385 (4)
C2—H2	0.9300	C19—H19	0.9300
C3—C4	1.511 (4)	C20—C21	1.384 (4)
C3—H3A	0.9700	C20—H20	0.9300
C3—H3B	0.9700	C21—C22	1.380 (4)
C4—C5	1.507 (3)	C21—H21	0.9300
C4—H4A	0.9700	C22—H22	0.9300
			
N7^i^—Cu1—N7	180.00 (18)	C6—C7—H7	119.8
N7^i^—Cu1—N1^i^	90.26 (8)	C9—C8—C7	120.4 (3)
N7—Cu1—N1^i^	89.74 (8)	C9—C8—H8	119.8
N7^i^—Cu1—N1	89.74 (8)	C7—C8—H8	119.8
N7—Cu1—N1	90.26 (8)	C8—C9—C10	119.7 (3)
N1^i^—Cu1—N1	180.00 (11)	C8—C9—H9	120.1
N8^ii^—Cu2—N8	180.00 (19)	C10—C9—H9	120.1
N8^ii^—Cu2—N4	89.33 (8)	C11—C10—C9	120.1 (2)
N8—Cu2—N4	90.67 (8)	C11—C10—H10	119.9
N8^ii^—Cu2—N4^ii^	90.67 (8)	C9—C10—H10	119.9
N8—Cu2—N4^ii^	89.33 (8)	C10—C11—C6	121.2 (2)
N4—Cu2—N4^ii^	180.00 (16)	C10—C11—H11	119.4
C1—N1—C2	102.3 (2)	C6—C11—H11	119.4
C1—N1—Cu1	129.55 (16)	N6—C12—N4	114.5 (2)
C2—N1—Cu1	128.06 (16)	N6—C12—H12	122.8
C2—N2—N3	102.5 (2)	N4—C12—H12	122.8
C1—N3—N2	109.74 (19)	N4—C13—N5	109.8 (2)
C1—N3—C3	129.3 (2)	N4—C13—H13	125.1
N2—N3—C3	120.9 (2)	N5—C13—H13	125.1
C13—N4—C12	103.1 (2)	N5—C14—C15	110.65 (18)
C13—N4—Cu2	126.30 (16)	N5—C14—H14A	109.5
C12—N4—Cu2	130.52 (16)	C15—C14—H14A	109.5
C13—N5—N6	110.04 (19)	N5—C14—H14B	109.5
C13—N5—C14	128.9 (2)	C15—C14—H14B	109.5
N6—N5—C14	121.02 (19)	H14A—C14—H14B	108.1
C12—N6—N5	102.6 (2)	C16—C15—C14	112.52 (18)
C23—N7—Cu1	169.1 (2)	C16—C15—H15A	109.1
C24—N8—Cu2	163.5 (2)	C14—C15—H15A	109.1
N3—C1—N1	110.6 (2)	C16—C15—H15B	109.1
N3—C1—H1	124.7	C14—C15—H15B	109.1
N1—C1—H1	124.7	H15A—C15—H15B	107.8
N2—C2—N1	114.9 (2)	O2—C16—C17	120.8 (2)
N2—C2—H2	122.6	O2—C16—C15	120.6 (2)
N1—C2—H2	122.6	C17—C16—C15	118.55 (19)
N3—C3—C4	110.8 (2)	C18—C17—C22	119.4 (2)
N3—C3—H3A	109.5	C18—C17—C16	119.2 (2)
C4—C3—H3A	109.5	C22—C17—C16	121.4 (2)
N3—C3—H3B	109.5	C19—C18—C17	120.4 (2)
C4—C3—H3B	109.5	C19—C18—H18	119.8
H3A—C3—H3B	108.1	C17—C18—H18	119.8
C5—C4—C3	113.0 (2)	C18—C19—C20	120.1 (3)
C5—C4—H4A	109.0	C18—C19—H19	120.0
C3—C4—H4A	109.0	C20—C19—H19	120.0
C5—C4—H4B	109.0	C21—C20—C19	120.1 (2)
C3—C4—H4B	109.0	C21—C20—H20	120.0
H4A—C4—H4B	107.8	C19—C20—H20	120.0
O1—C5—C6	120.9 (2)	C22—C21—C20	120.3 (3)
O1—C5—C4	120.6 (2)	C22—C21—H21	119.8
C6—C5—C4	118.5 (2)	C20—C21—H21	119.8
C7—C6—C11	118.1 (2)	C21—C22—C17	119.7 (2)
C7—C6—C5	123.3 (2)	C21—C22—H22	120.2
C11—C6—C5	118.6 (2)	C17—C22—H22	120.2
C8—C7—C6	120.4 (2)	N7—C23—S1	178.5 (2)
C8—C7—H7	119.8	N8—C24—S2	179.5 (3)
			
N7^i^—Cu1—N1—C1	−34.9 (2)	C11—C6—C7—C8	0.3 (4)
N7—Cu1—N1—C1	145.1 (2)	C5—C6—C7—C8	−179.5 (2)
N7^i^—Cu1—N1—C2	149.3 (2)	C6—C7—C8—C9	0.8 (4)
N7—Cu1—N1—C2	−30.7 (2)	C7—C8—C9—C10	−0.9 (4)
C2—N2—N3—C1	0.7 (3)	C8—C9—C10—C11	−0.1 (4)
C2—N2—N3—C3	−176.3 (3)	C9—C10—C11—C6	1.2 (4)
N8^ii^—Cu2—N4—C13	157.5 (2)	C7—C6—C11—C10	−1.3 (4)
N8—Cu2—N4—C13	−22.5 (2)	C5—C6—C11—C10	178.5 (2)
N8^ii^—Cu2—N4—C12	−27.2 (2)	N5—N6—C12—N4	0.7 (3)
N8—Cu2—N4—C12	152.8 (2)	C13—N4—C12—N6	−0.3 (3)
C13—N5—N6—C12	−0.9 (3)	Cu2—N4—C12—N6	−176.39 (19)
C14—N5—N6—C12	−179.9 (2)	C12—N4—C13—N5	−0.3 (3)
N1^i^—Cu1—N7—C23	117.2 (11)	Cu2—N4—C13—N5	176.02 (16)
N1—Cu1—N7—C23	−62.8 (11)	N6—N5—C13—N4	0.8 (3)
N4—Cu2—N8—C24	−146.7 (8)	C14—N5—C13—N4	179.7 (2)
N4^ii^—Cu2—N8—C24	33.3 (8)	C13—N5—C14—C15	−107.1 (3)
N2—N3—C1—N1	−0.7 (3)	N6—N5—C14—C15	71.7 (3)
C3—N3—C1—N1	176.0 (2)	N5—C14—C15—C16	−172.76 (19)
C2—N1—C1—N3	0.4 (3)	C14—C15—C16—O2	2.3 (3)
Cu1—N1—C1—N3	−176.23 (17)	C14—C15—C16—C17	−177.49 (19)
N3—N2—C2—N1	−0.5 (4)	O2—C16—C17—C18	0.7 (3)
C1—N1—C2—N2	0.1 (3)	C15—C16—C17—C18	−179.5 (2)
Cu1—N1—C2—N2	176.8 (2)	O2—C16—C17—C22	179.9 (2)
C1—N3—C3—C4	143.1 (3)	C15—C16—C17—C22	−0.3 (3)
N2—N3—C3—C4	−40.5 (3)	C22—C17—C18—C19	−0.1 (3)
N3—C3—C4—C5	−166.5 (2)	C16—C17—C18—C19	179.1 (2)
C3—C4—C5—O1	5.0 (3)	C17—C18—C19—C20	0.8 (4)
C3—C4—C5—C6	−176.4 (2)	C18—C19—C20—C21	−0.6 (4)
O1—C5—C6—C7	174.5 (2)	C19—C20—C21—C22	−0.2 (4)
C4—C5—C6—C7	−4.1 (3)	C20—C21—C22—C17	0.9 (4)
O1—C5—C6—C11	−5.3 (3)	C18—C17—C22—C21	−0.8 (3)
C4—C5—C6—C11	176.0 (2)	C16—C17—C22—C21	−179.9 (2)

Symmetry codes: (i) −*x*, −*y*+1, −*z*; (ii) −*x*+1, −*y*, −*z*.

### Hydrogen-bond geometry (Å, º)

**Table:**

*D*—H···*A*	*D*—H	H···*A*	*D*···*A*	*D*—H···*A*
C13—H13···O1	0.93	2.58	3.330 (3)	138

## References

[bb1] Bruker (2007). *APEX2* and *SAINT* Bruker AXS Inc., Madison, Wisconsin, USA.

[bb2] Guo, J.-H. & Cai, H. (2007). *Acta Cryst.* E**63**, m1322–m1324.

[bb3] Sheldrick, G. M. (1996). *SADABS* University of Göttingen, Germany.

[bb4] Sheldrick, G. M. (2008). *Acta Cryst.* A**64**, 112–122. 10.1107/S0108767307043930 18156677

[bb5] Yue, Y.-F., Gao, E.-Q., Fang, C.-J., Zheng, T., Liang, J. & Yan, C.-H. (2008). *Cryst. Growth Des.* **9**, 3295–3301.

